# Measurement of empathy among health professionals during Syrian crisis using the Syrian empathy scale

**DOI:** 10.1186/s12909-021-02835-0

**Published:** 2021-07-29

**Authors:** Mayssoon Dashash, Mounzer Boubou

**Affiliations:** 1 Faculty of Dentistry, Damascus University, Damascus, Syria; 2 Program of Medical Education, Syrian Virtual University, Damascus, Syria; 3 Faculty of Education, Tishreen University, Latakia, Syria

**Keywords:** Attitude, Empathy, Measurement, Syria, Health professional, Student

## Abstract

**Background:**

Health professionals should have certain degree of empathy to eliminate the pain and suffering of their patients. There is a need to design a scale, which can assess empathy among health professionals and is relevant to community and culture. Therefore, this study was undertaken to measure the empathy among Syrian health professionals and students of health professions using a newly designed Syrian Empathy Scale that is relevant to community during Syrian crisis.

**Methods:**

A cross-sectional observational study was undertaken. A total of 214 participants (118 males and 96 females) responded to the Syrian Empathy Scale SES from Medical (*n* = 62), Dental (*n* = 152). They were 59 undergraduates, 116 postgraduates and 39 general practitioners. The SES was designed as a tool that includes 20 items in a 7-point Likert-type scale with overall score ranges from 20 to 140. Group comparisons of the empathy scores were conducted using *t*-test and analysis of variance (ANOVA). A factor analysis was performed. Bartlett’s test of the sphericity and the KMO measure of sampling adequacy were also determined. Cronbach’s alpha was calculated.

**Results:**

A significant difference was found between males and females in the SES mean score. The ANOVA analysis showed that the SES empathy scores of dentists were higher than the SES empathy scores in medical doctors with no significant difference. The SES empathy score of undergraduates was significantly higher than postgraduates and practitioners. Findings of KMO indicated sampling adequacy (KMO = 0.824 > 0.7) and the value of Bartlett’s test of the sphericity (1255.65, df = 190, *P-*value< 0.001) proved that the factor analysis is meaningful and acceptable. The results of varimax rotation proved that five main factors were retained.

**Conclusion:**

Findings of this study support the reliability of the newly designed Syrian Empathy Scale for measuring empathy in the field of health care. The SES can be suggested for assessing empathy in different health educational programs. However, future works are still essential to support the validity of the scale as well as to ascertain the role of empathy in improving health care.

## Background

Attitude which has three components including cognitive (what we think and believe), affective (what we feel and experience) and behavioral (what we do), has been a good predictor of professional behavior and clinical competence [1].

Empathy is one of the component of attitude that enables health professionals to understand the experience of patient, concerns and perspectives [2]. It includes the ability and capacity of the doctor to see the world from the perspective of the patient and to walk himself in the patient’s shoes, without interfering with the professional responsibilities and obligations [3]. Health professionals should have certain degree of empathy and should put their knowledge, skills and attitude in their clinical practice to eliminate the pain and suffering of their patients [4].

Previous studies have found that empathy towards patients can positively affect patient satisfaction, compliance and clinical outcome [5] and that patients of health professionals who achieved well in empathy measurement had better control of their disease and better prognosis when compared to patients of physicians with lower empathy scores [6]. Researchers have addressed the need to investigate empathy in terms of direction (favorable/ unfavorable), intensity (positive, negative), and range of feeling (pervasiveness), consistency, and salience. They have also addressed the need to measure empathy either at admission to medical school or during clinical training [7–10]. However, research and measurement of empathy remained limited due to the lack of clarity in its conceptualization and lack of standardized tool that can measure it [11].

To measure empathy, it is important to have a consensus definition of it as a multidimensional construct and to understand its cognitive and emotional components [12]. Emotional empathy with its three subdivisions “emotion contagion, proximal and peripheral responsivity” [13], is the reaction to the response of others [14], experience their feelings, emotions, and sharing their emotional experiences [12]. Cognitive empathy with its subdivisions “perspective taking, and online simulation*”* is the process of understanding the perspective of another person, the capacity to judge, understand the intentions of others and consequently help them [12, 15]. Researchers have addressed the importance of both components of empathy in clinical outcome [16]. Some researchers have indicated that cognitive empathy is more prominent in medical setting than emotional empathy [16]. Others indicated that emotional empathy could be useful to a limited extent but could also affect the clinical decision if it is in excess as it can create fatigue and exhaustion [16]. In this regard, it is important to design a scale that is relevant to community and culture, which can measure cognitive and emotional empathy among health professionals and students.

During Syrian crisis, the role of Syrian health professional as a “human rather than a machine” should be emphasized in order to respond to health, psychological and social needs of patients who suffer from different economic, social, psychological, and health problems during crisis [17–20]. The development of a valid precise tool that enables the measurement of empathy and comparison with other societies can be of particular importance. The measurement should be carried out using a reliable, valid, effective, simple, and understandable scale [21].

Previous work has emphasized the need to use variant methods for measurement such as direct self-report questionnaires, paper cases and observation of behavior [17]. The present study aimed at measuring cognitive and emotional empathy among Syrian health professionals and students of health professions using the Syrian Empathy Scale SES.

## Methods

### Participants

This is a cross-sectional, observational study conducted among Syrian health professionals in August 2020. It was performed in accordance with the Declaration of Helsinki and was approved by the ethical committee of the Faculty of Dentistry, in Damascus University(No.561/s). The data collection was performed through uploading an on-line survey and distributing the SES on all Syrian health webs and social media. A total of 214 participants (118 males and 96 females) enrolled in this study from Medical (62), and Dental faculties (152). They were 116 undergraduates, 59 postgraduates and 39 general practitioners. Informed consent was taken assuring the anonymity and confidentiality of the answers.

### Instrument of measurement

The Syrian Empathy Scale was developed by MD in the Faculty of Dentistry, Damascus University to assess the empathy among health professionals during Syrian crisis. All attitude statements were designed to be simple, clear and belonged to the same attitude variable in order to decrease the wrong interpretation of the results. To increase the validity and reliability of statements, three of academic members were asked to test the clarity and the relevance of statements in the light of the aim of the study and to define whether the statement is reflecting the cognitive or emotional state [22]. Modifications and suggestions were considered with no deletion of any of the items. For internal consistency, Pearson correlation coefficient was calculated for all items and all values of correlations were significant at values (*P* = 0.05). In addition, about half of the items [20] were negatively written [23] in which scores would range between 20 and 140, and higher values would indicate a higher degree of empathy [24]. Scoring was reversed for negative items in order to obtain the same direction of positive items [1]. A Likert-scale, which is one-dimensional and non-comparative scaling technique [25], has been used to determine the extent, to which the health professionals and students would agree or disagree with the statement in which quantitve data can be obtained [26]. To add additional granularity [25], a 7-point rather than 5-point scale ranged from 1 = Strongly Disagree, 2: Disagree, 3: Slightly disagree, 4: Undecided, 5: Slightly agree, 6: Agree, 7: Strongly agree has been implemented [27]. Table 1 shows the designed scale together with the nine cognitive and 11 emotional empathy statements.

**Table 1:** The Syrian Empathy Scale: Number and percentages of responses in cognitive and emotional questions

items	strongly agree	agree	partially agree	not decided	partially disagree	disagree	strongly disagree
**Q1: It is of great value to have right away sense of empathy towards ill people (emotional)**	9 (4%)	11 (5%)	16 (7%)	55 (26%)	44 (21%)	34 (16%)	45 (21%)
**Q2: I can quickly feel the pain of the poor patient regardless of their (social, health, religious background. (emotional)**	6 (3%)	2 (1%)	10 (5%)	28 (13%)	42 (20%)	52 (24%)	74 (35%)
**Q3: It is important to recognize the feeling of heart broken patients and put yourself at their place (cognitive)**	9 (4%)	6 (3%)	6 (3%)	19 (9%)	31 (14%)	57 (27%)	86 (40%)
**Q4: I consider that understanding the background and culture of my patient is very important to make treatment successful(cognitive)**	6 (3%)	6 (3%)	9 (4%)	16 (7%)	27 (13%)	37 (17%)	113 (53%)
**Q5: I try to connect with my patient’s pain to help him/ her feel supported(cognitive)**	23 (11%)	16 (7%)	16 (7%)	23 (11%)	36 (17%)	42 (20%)	58 (27%)
**Q6: Being involved in patient feeling can greatly improve clinical outcome (emotional)**	15 (7%)	14 (7%)	16 (7%)	33 (15%)	43 (20%)	50 (23%)	43 (20%)
**Q7: My patients have better satisfaction and control of their disease when I understand their feeling and emotional state (cognitive)**	6 (3%)	9 (4%)	7 (3%)	25 (12%)	56 (26%)	58 (27%)	53 (25%)
**Q8: Being open, encouraging and warm with my patient will increase compliance, satisfaction and improve health (emotional)**	4 (2%)	4 (2%)	9 (4%)	22 (10%)	37 (17%)	65 (30%)	73 (34%)
**Q9: Viewing the world from the perspective of the patient would improve doctor-patient relationship and improve the clinical outcome (cognitive)**	7 (3%)	7 (3%)	6 (3%)	22 (10%)	43 (20%)	49 (23%)	80 (37%)
**Q10 Patient talk to me about their personal problems as I try to understand their suffering (cognitive)**	14 (7%)	17 (8%)	21 (10%)	33 (15%)	39 (18%)	38 (18%)	52 (24%)
**Q11: It is not important to know patient background and culture in order to provide effective treatment (cognitive)**	77 (36%)	40 (19%)	26 (12%)	17 (8%)	16 (7%)	20 (9%)	18 (8%)
**Q12: Paying attention to patient’ feeling during history taking might negatively affect professional responsibilities(emotional)**	29 (14%)	38 (18%)	30 (14%)	44 (21%)	37 (17%)	14 (7%)	22 (10%)
**Q13: Physicians should not become emotionally involved in the patients’ suffering as this might have bad effects(emotional)**	23 (11%)	25 (12%)	42 (20%)	53 (25%)	25 (12%)	14 (7%)	32 (15%)
**Q14: I am not interested in patient’s personal matters as these are not relevant to medical treatment(emotional)**	40 (19%)	42 (20%)	41 (19%)	36 (17%)	22 (10%)	14 (7%)	19 (9%)
**Q15: Viewing things from patient’ perspectives might confuse me and make me too distracted to take the right clinical decision(cognitive)**	42 (20%)	35 (16%)	31 (14%)	43 (20%)	26 (12%)	14 (7%)	23 (11%)
**Q16: Patient compliance and effective medical treatment are the only factors that can affect clinical outcome rather than talking to patients about their problems (emotional)**	46 (21%)	42 (20%)	45 (21%)	31 (14%)	17 (8%)	15 (7%)	18 (8%)
**Q17: Response to patient needs might affect clinical decision(emotional)**	22 (10%)	27 (13%)	29 (14%)	37 (17%)	41 (19%)	31 (14%)	27 (13%)
**Q18: Empathy towards patients makes me burned out and makes me feel emotionally exhausted (emotional)**	36 (17%)	33 (15%)	22 (10%)	30 (14%)	33 (15%)	25 (12%)	35 (16%)
**Q19: Being involved with patient feeling is not important to provide better care (emotional)**	88 (41%)	50 (23%)	19 (9%)	21 (10%)	11 (5%)	11 (5%)	14 (7%)
**Q20: My understanding to the good doctor is the one who provides the best diagnosis and treatment regardless of the relationship with the patients (cognitive)**	46 (21%)	41 (19%)	30 (14%)	33 (15%)	9 (4%)	16 (7%)	39 (18%)

*Scoring was reversed for negative statements

### Statistical analysis

Statistical analysis was performed in SPSS Version 25 (SPSS Inc., Chicago, IL, USA). The descriptive statistics was applied. Mean and standard deviation (SD) together with frequencies and percentages of students in the light of their specialization and gender were calculated [9]. The SES mean score was categorized according to gender, specialization, level of practice. The mean score for all participants and the sum of cognitive and affective empathy scores [22] were also measured. Number and percentages of responses for each level of agreement in each item were also determined. Group comparisons of the empathy scores were conducted using *t*-test to determine the significance difference between males and females in the empathy mean scores. Analysis of variance (ANOVA) was carried out to determine the significance differences between the level of practices and specialization [25]. *P* < 0.05 was considered as the significant level. The internal consistency of SES was 0.85. Second, a factor analysis was performed following the next steps: (a) Bartlett’s test of the sphericity and the KMO (Kaiser Meyer Olkin) measure of sampling adequacy were determined to measure the goodness of factor analysis. (b) The principal component analysis was performed to extract the number of components. (c) The retained components were submitted to a varimax rotation and the criteria of eigenvalue > 1 six main factors were retained. In addition, factor coefficients greater than 0.4 were used to make the interpretation of suggested components. To analyze the internal consistency of these factors the test of alpha Cronbach was used.

## Results

The final sample was composed of 214 participants. There were 96 females (45%) and 118 males (55%), from medical (62) and dental specializations (152) and they distributed as follows: 116(54%) undergraduates, 59(28%) postgraduates, and 39 (18%) general practitioners. The Descriptive analysis indicated that the mean value of the SES was 98.12 ± 18.076, the minimum score was 32, and the maximum value was 137. The skewness and kurtosis of the SES was −.587 ± .166 and 1.010 ± .331 respectively.

Regarding item statistics, participants used the full range of responses for all items. Table 1 presents the number and frequencies of all items together with the item mean scores which ranged from 3.83 for item 17 “Response to patient needs might affect clinical decision” to 5.90 for item 4 “I consider that understanding the background and culture of my patient is very important to make treatment successful”.

**Fig. 1:**
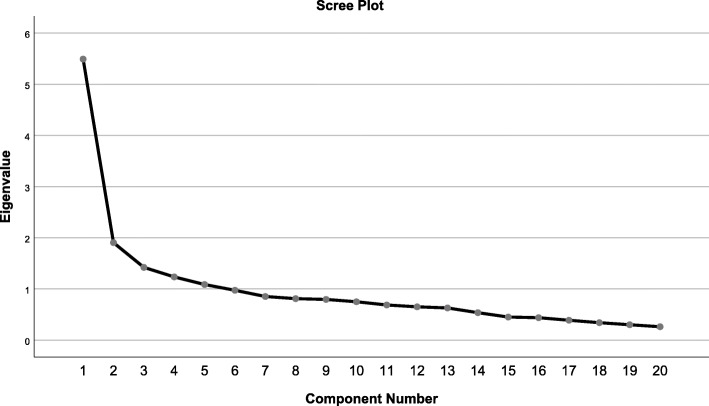
The eigenvalues scree plot

The summary results of factor analysis for the 20 items of SES are reported in Table 2. It presents the retained extracted five components, initial Eigenvalues, percentages of variance, and cumulative percentages. Figure 1 presents the eigenvalues scree plot.

**Table 2:** findings of factor analysis

Component	Initial Eigenvalues		
	Total	% of Variance	Cumulative %
1	5.513	27.565	27.565
2	1.914	9.569	37.135
3	1.404	7.021	44.155
4	1.243	6.214	50.369
5	1.110	5.548	55.917

Each factor had eigenvalue greater than one, accounting for 55.92% of a total variation before rotation.

Findings of KMO indicated sampling adequacy (KMO = 0.824 > 0.7) and the value of Bartlett’s test of the sphericity (1255.65, df = 190, *P*-value< 0.001) proved that the factor analysis is meaningful and acceptable [28]. In addition, the reliability analysis of internal factors calculated showed a high internal consistency since the Cronbach’s alpha value (0.85) was greater than 0.5 for all factors except factor 5, which was composed by two items. The composition of different factors is analyzed considering the items associated, with a value greater than 0.4. The results of varimax rotation are presented in Table 3. Five main factors were retained (eigenvalue > 1). Factor 1, which accounted for 14.715 of the variance, was labeled as “Care and Understanding” based on the contents of (items 7, 8, 6, 10, 5, 4, 9). Factor 2, which accounted for 12.286 of the variance, was labeled as “Feeling” based on the contents of [1–3]. Factor 3, which accounted for 11.686 of the variance, was labeled as “Health Care” based on the contents [11, 14, 16, 19, 20]. Factor 4, which accounted for 9.368 of the variance, “Negative Empathy Impact”, based on the contents of [12, 13, 18].Factor 5, which accounted for 7.862 of the variance was labeled as “Clinical Decision Making”, based on the contents of [15, 17].

**Table 3:** Results of varimax rotation

Rotated Component Matrix	Component				
	1	2	3	4	5
Care and Understanding					
7. يشعر مرضاي بالرضى ويسيطرون على آلامهم عندما أتفهم حالتهم النفسية والعاطفية 7. My patients have better satisfaction and control of their disease when I understand their feeling and emotional state (cognitive)	0.772				
8. يزيد انفتاحي وتشجيعي لمرضاي وكلماتي الدافئة من تجاوبهم ورضاهم وتحسين صحتهم 8. Being open, encouraging and warm with my patient will increase compliance, satisfaction and improve health (emotional)	0.703				
6. الانخراط بحياة وإحساس المريض من النتيجة السريرية والشفاء بصورة كبيرة يحسن 6. Being involved in patient feeling can greatly improve clinical outcome (emotional)	0.686				
10. يصارحني المرضى عن مشاكلهم الشخصية لأني أحاول أن أتفهم معاناتهم 10. Patient talk to me about their personal problems as I try to understand their suffering (cognitive)	0.672				
5. أحب أن أشعر بألم مريضي لأشعره بأني داعم له 5. I try to connect with my patient’s pain to help him/ her feel supported(cognitive)	0.552			0.347	
4. والثقافية أمر بالغ الأهمية للحصول على معالجة ناجحة أنا أشعر بأن فهم البيئة الاجتماعية والاقتصادية 4. I consider that understanding the background and culture of my patient is very important to make treatment successful(cognitive)	0.427		0.414		
9. النظر إلى الأمور من وجهة نظر المرضى يمكن أن يحسن علاقة مريض- طبيب ويحسن الوضع السريري للمريض 9. Viewing the world from the perspective of the patient would improve doctor-patient relationship and improve the clinical outcome (cognitive)	0.341	0.328			
Feeling					
1. من الأهمية بمكان خلال ممارستك أن يكون لديك شعور فوري بالتعاطف تجاه المرضى 1. It is of great value to have right away sense of empathy towards ill people (emotional)		0.813			
3. من المهم أن تحس بشعور المرضى المنكسرين وتضع نفسك في مكانهم. 3. It is important to recognize the feeling of heart broken patients and put yourself at their place (cognitive)		0.764			
2. أستطيع أن أشعر بسرعة بألم المريض الفقير بصرف النظر عن وضعه الاجتماعي والاقتصادي والصحي والديني 2. I can quickly feel the pain of the poor patient regardless of their (social, health, religious background. (emotional)		0.758			
Health care					
11. ليس من الضروري تفهم البيئة الثقافية والاجتماعية للمرضى للحصول على معالجة فعالة 11. It is not important to know patient background and culture in order to provide effective treatment (cognitive)			0.787		
19. لا يعد الإحساس بمشاعر المريض من الأمور المهمة لتقديم الرعاية الصحية 19. Being involved with patient feeling is not important to provide better care (emotional)			0.666		
14. لا أهتم بالانخراط بالأمور الشخصية للمريض لانعدام أهمية ذلك بالمعالجة الطبية. 14. I am not interested in patient’s personal matters as these are not relevant to medical treatment(emotional)			0.578		
16. إن العوامل الوحيدة التي تؤثر على النتيجة السريرية هي استجابة المريض لخطة المعالجة وفعالية هذه المعالجة وليس التكلم مع المرضى عن مرضهم 16. Patient compliance and effective medical treatment are the only factors that can affect clinical outcome rather than talking to patients about their problems (emotional)			0.526		0.498
20. فهمي للطبيب الجيد هو ذلك الذي يقدم التشخيص والمعالجة بصورة جيدة وبغض النظر عن علاقته مع مرضاه. 20: My understanding to the good doctor is the one who provides the best diagnosis and treatment regardless of the relationship with the patients (cognitive)			0.382		0.340
Negative Impact of Empathy					
12. يمكن للاكتراث إلى مشاعر المريض خلال أخذ القصة السريرية أن يؤثر على الواجبات والمسؤوليات المهنية للطبيب 12. Paying attention to patient’ feeling during history taking might negatively affect professional responsibilities(emotional)				0.749	
13. يجب على الطبيب ألا ينخرط عاطفيا بمعاناة المريض لما لذلك من عواقب وتأثيرات سلبية 13. Physicians should not become emotionally involved in the patients’ suffering as this might have bad effects(emotional)				0.611	
18. يشعرني التعاطف مع المرضى بالإنهاك والاستنزاف العاطفي 18. Empathy towards patients makes me burned out and makes me feel emotionally exhausted (emotional)				0.544	
Clinical Decision Making					
17. اتخاذ القرار السريري المناسب قد تؤثر الاستجابة لاحتياجات المرضى على 17. Response to patient needs might affect clinical decision(emotional)					0.786
15. يؤدي النظر إلى الأشياء من وجهة نظر المريض إلى تشتيت انتباهي وتركيزي ومنعي من اتخاذ القرار السريري الصحيح 15. Viewing things from patient’ perspectives might confuse me and make me too distracted to take the right clinical decision(cognitive)			0.310	0.359	0.502
Variance	14.715	12.286	11.686	9.368	7.862
Alpha Cronbach	0.781	0.787	0.679	0.547	0.448

In addition, the Pearson correlation coefficients demonstrated positive and statistically significant correlations between each item score and the total score of the SES. The item total score correlations ranged from 0.489 to a high of 0.864.

In addition, the item-total correlations for each factor are presented in Table 4.

**Table 4:** The item-total correlations for each factor

Factor		Item4	item5	Item6	Item7	Item8	Item9	Item10
Care and Understanding	Pearson Correlation	.489^**^	.675^**^	.722^**^	.755^**^	.692^**^	.583^**^	.705^**^
	Sig. (2-tailed)	0.000	0.000	0.000	0.000	0.000	0.000	0.000
		item1	item2	item3				
Feeling	Pearson Correlation	.864^**^	.794^**^	.853^**^				
	Sig. (2-tailed)	0.000	0.000	0.000				
		item11	item14	item16	item19	item20		
Health care	Pearson Correlation	.680^**^	.700^**^	.631^**^	.704^**^	.609^**^		
	Sig. (2-tailed)	0.000	0.000	0.000	0.000	0.000		
		item12	item13	item18				
Negative Empathy Impact	Pearson Correlation	.704^**^	.743^**^	.730^**^				
	Sig. (2-tailed)	0.000	0.000	0.000				
		item15	item17					
Clinical Decision Making	Pearson Correlation	.810^**^	.795^**^					
	Sig. (2-tailed)	0.000	0.000					

A significant difference was found between males and females in the SES mean score. The empathy score of female students (mean = 102.36; SD = 15.28) was significantly higher than the scores of the male students (Mean = 94.67, SD = 19.68; *t* = 3.14, *P* = 0.002). Moreover, no significant difference was found between medical doctors and dentists in the SES mean score. The SES empathy scores of medical students (mean = 95.55; SD = 22.99) were lower than the scores of the dental students with no significant difference (Mean = 99.17, SD = 15.83; t = 1.36, *P* = 0.259).

In addition, the ANOVA analysis showed that the SES empathy score of practitioners (mean ± SD 101.00 ± 17.31) was significantly (F = 3.74, *P* = 0.025) higher than undergraduates and postgraduates (mean ± SD 99.91 ± 17.51, 92.71 ± 19.29 respectively).

To discriminate between professionals and students in each specialization, the ANOVA analysis was also performed. Findings showed significant difference between dental undergraduate students, postgraduate dental students and general dental practitioners in the SES mean score (F = 4.2, *P* = 0.017). The SES empathy scores of postgraduates (mean = 92.57; SD = 15.89) were significantly lower than the scores of dental undergraduates (mean = 100.79; SD = 15.08) and general dental practitioners (mean = 102.13, SD = 16.21). However, no significant difference between medical undergraduate students (mean = 97.36; SD = 23.24), postgraduate medical students (mean = 92.91; SD = 23.75) and general medical practitioners (mean = 96.62; SD = 21.77) was found in the SES mean score (F = 0.253, *P* = 0.777).

Table 5 represents the result of Least Significant Difference LSD test and a significant difference between undergraduates and postgraduates (*P* = 0.013) in the SES mean score as well as the difference between undergraduates and practitioners (*P* = 0.027).

**Table 5:** The differences between undergraduates, postgraduates and general practitioners

(I) Professional situation	(J) Professional situation	Mean Difference (I-J)	Sig.
Specialization level	undergraduates	−7.193-^*^	0.013
	general practitioners	−8.288-^*^	0.027
Undergraduates	Specialization level	7.193^*^	0.013
	general practitioners	−1.094	0.743
General practitioners	Specialization level	8.288^*^	0.027
	undergraduates	1.094	0.743

Moreover, testing the SES mean score of participants according to their gender and specialization using multivariate analysis has indicated that there is no effect (F = 0.04, *P* = 0.842). In addition, testing the SES mean score of participants according to their gender and level of practice using multivariate analysis has indicated that there is no effect (F = 0.31, *P* = 0.735).

## Discussion

Given the importance of teaching attitude to medical students, questions have been raised regarding the possibilities of finding a standardized, valid reliable and feasible instrument that can measure it [17]. Attitude is a complex construct. The decision should be made whether the instrument would test cognitive, psychomotor or affective aspects [26].

Empathy is one of the component of attitude that enables health professionals to understand the experience of patient, concerns and perspectives [2].

Previous studies have indicated that empathy is not well covered in medical curricula [17–20]. Researchers have addressed the need to measure empathy either at admission to medical school or during clinical training [7, 8, 10]. Health professionals should have certain degree of empathy and should put their knowledge, skills and attitude in their clinical practice to eliminate the pain and suffering of their patients [4].

The Jefferson Scale of Empathy (JSE) has been used to evaluate empathy among health professionals and students of health professions in several countries such as the USA, Poland, Korea, Italy, Japan. It has been standardized for its validity and reliability [3]. However, no empathy scales have been designed to measure the empathy of health professionals who are located in an area exposed to war and are practicing medicine in regions with conflicts. Only one recent study has investigated the attitude of host countries’ citizens toward refugee children [29]. This study has addressed the importance of designing, developing and validating scales that measure attitudes in fragile areas in which people may suffer from violence, internal displacement and adverse psychological environment.

In Syria, people after ten years of war are suffering from gross human rights violations, international sanction, shortage of medicine and medical equipments, chronic hunger, and the COVID-19 pandemic. In these situations, health professionals should provide the life-saving assistance to community; respond to health, psychological and social needs of patients who suffer from different economic, social, psychological, and health problems regardless of their own daily suffering and daily miserable conditions. Therefore, the Syrian Empathy Scale SES was developed to measure the empathy in the context of Syrian health professionals during the crisis, support decision-making processes, and help identifying areas that require further attention and training.

The designed scale includes 20 questions and the overall score ranges from twenty to one hundred and forty in which higher scores indicate a better empathic relationship in the medical and therapeutic care.

The SES was designed to be simple, cheap, readable and practical useful tool that can be used in practice settings as an attempt to shed some light on the role of Syrian health professionals during conflict, with respect to health care, understanding, feeling and clinical decision-making.

Writing statement, which is a crucial part in designing the empathy scale to anonymous group [17, 24], has not been an easy task as it has to be simple, short, direct debatable, clear-cut, meaningful and interesting. Attempt was made to make statements understandable and belonged to the same attitude variable as well as to make them relevant to the community during Syrian crisis [24]. For instance, issues such as feeling the pain of poor patients regardless of their social, health, and religious background (item 2) as well as recognizing the feeling of heart broken patients, (items 3) have enabled us to assess the cognitive and emotional attitude of health professionals in conflicts. However, the comparison between the SES and other designed scales has been inappropriate and testing the convergent validity would be not suitable due to differences existed in the constructs.

After the factorial analysis, it was possible to identify five different components of empathy (*Care and Understanding, Feeling, Health Care, Negative Empathy Impact and Clinical Decision Making*). The findings support the goodness of the factorial analysis. Duarte et al. identified 6 components of empathy through the factorial analysis (*compassionate care, perspective taking, cognitive dimension, standing in patient shoes clinical outcomes, no influence by others*) and could also supported the goodness of the analysis [3].

To increase the reliability of measurement, decrease error and save time, attempts was made to make each statement has one interpretation, contains one complete thought and one specific attitude related to one issue [24]. Likert scales was also adopted in order to identify the extent to which the respondent would agree or disagree with the object [26]. Negatively wording of half of the attitude statements was applied to provide a true measurement of an attitude, avoid the acquiescence bias and minimize extreme response that might be caused because of some respondents who might tend to agree with most statements [23]. Moreover, careful statistical methods and analysis such as Cronbach’s alpha reliability coefficient were applied in order to verify the internal consistency of the applied scales [23]. The value of Cronbach’s alpha which were considered as good (0.85) provided evidence about the reliability of the applied scale [30]. The alpha coefficient obtained was similar to other values obtained in some studies [31, 32] and was higher than the values obtained in other studies [2, 33, 34]. The values of item-total correlations obtained for each item was higher than 0.48 indicating that an item was related to the overall scale. Anonymous questionnaires to a sufficient sample size was considered in order to further validate and improve the designed scale [13]. Accordingly, this questionnaire can be considered as reliable for measuring empathy among Syrian health professionals.

The findings of the present study showed that the SES empathy score of undergraduates was significantly higher than postgraduates and it was higher in dental specialization (100.79) when compared to medicine(97.36). Similar findings were reported about the decline in empathy with increasing age or year of education [35–37]. Studies have attributed many factors to this consistent finding. The stress of academic performance, long work hours [38], lack of quality sleep, and increased responsibilities with age [39] are some factors that contribute to declining empathy among older individuals [40]. Further studies, using the SES scale, with a larger samples size are still needed to ascertain our findings.

The present study reported a significant difference between males and females in the SES mean score and higher empathy scores among females. The findings were consistent with previous findings reported [41, 42] who attributed this to qualitative variance in integrating emotional information between males and females genders that can affect the decision-making process [40]. Similarly, Hojat et al. attributed this to social learning, genetic predisposition, and evolutionary underpinnings [43].

The SES has been a great tool for assessing Empathy of Syrian health professionals However, several procedures are still essential to increase its validity and reliability before applying it in linguistically and culturally diverse settings. For instance, multiple tests and items such as questionnaires, papers cases and observation of behavior could be developed [17]. In addition, observation of medical students, during management of patients, can also be used together with empathy scale in order to improve the validity and reliability of the scale. An objective approach in which students are required to take OSCEs by standardized patients could also be suggested to explore the association between empathy scores and ratings of clinical competence in OSCE stations [7, 44–46].

## Conclusions

This study is the first of its kind in Syria that addressed the importance of empathy in the field of health care and the need of measuring it among health professionals and students of health professions. Findings of this study support the reliability of the newly designed Syrian Empathy Scale for measuring empathy in the field of health care. Our work is still in progress in order to combine our designed tool with qualitative investigation in order to explore the lived experience of Syrian health professionals and investigate areas that require further attention. This would further improve understanding about the role of empathy in improving health care and would support decision-making processes in identifying areas that require further attention and training.

## Data Availability

The datasets used and/or analyzed during the present study are available from the corresponding author upon a reasonable request.

## Abbreviations

SES: Syrian Empathy Scale
KMO: Kaiser Meyer Olkin
JSE: The Jefferson Scale of Empathy
LSD: Least Significant Difference
MD: Mayssoon Dashash
MB: Mounzer Boubou
SD: standard deviation
